# Effect of sulfasalazine on endothelium-dependent vascular response by the activation of Nrf2 signalling pathway

**DOI:** 10.3389/fphar.2022.979300

**Published:** 2022-10-24

**Authors:** Muhammed Ikbal Sonmez, Andleeb Shahzadi, Cagla Kose, Haktan Sonmez, Sibel Ozyazgan, Ahmet Gokhan Akkan

**Affiliations:** ^1^ Institute of Experimental Pharmacology and Toxicology, University Medical Center Hamburg-Eppendorf, Hamburg, Germany; ^2^ Department of Medical Pharmacology, Cerrahpasa Medical Faculty, Istanbul University-Cerrahpasa, Istanbul, Turkey; ^3^ DZHK (German Center for Cardiovascular Research), Partner Site Hamburg/Kiel/Lübeck, Hamburg, Germany; ^4^ Department of Medical Pharmacology, Medical Faculty, Halic University, Istanbul, Turkey; ^5^ Department of Medical Pharmacology, Medical Faculty, Bezmialem Vakif University Hospital, Istanbul, Turkey

**Keywords:** diabetes mellitus, sulfasalazine, Nrf2, ERK, rat aorta

## Abstract

**Background:** Diabetes mellitus leads to endothelial dysfunction and accumulation of oxygen radicals. Sulfasalazine-induced Nrf2 activation reduces oxidative stress in vessels. Thus, in the present study, we investigated the effects of sulfasalazine on endothelial dysfunction induced by high glucose. We also ascribed the underlying mechanism involved in glucose-induced endothelial dysfunction.

**Methods:** For this experiment we used 80 Wistar Albino rats thoracic aorta to calculate the dose response curve of noradrenaline and acetylcholine. Vessels were incubated in normal and high glucose for 2 h. To investigate glucose and sulfasalazine effects the vessels of the high glucose group were pre-treated with sulfasalazine (300 mM), JNK inhibitor (SP600125), and ERK inhibitor (U0126) for 30 min. The dose response curve was calculated through organ bath. The eNOS, TAS, TOS, and HO-1 levels were estimated by commercially available ELISA kits.

**Results:** In the high glucose group, the E_max_ for contraction was significantly higher (*p* < 0.001), and E_max_ for relaxation was lower than that of control. These functional changes were parallel with the low levels of eNOS (*p* < 0.05). High glucose vessel treated with sulfasalazine showed low E_max_ value for contraction (*p* < 0.001) however, the E_max_ for relaxation was significantly high (*p* < 0.001) when compared to high glucose group. In the JNK group, E_max_ for contraction and relaxation was inhibited (*p* < 0.001) compared to sulfasalazine treated vessels. HO—1 enzyme levels were significantly low (*p* < 0.01) with sulfasalazine but higher with ERK inhibitor (*p* < 0.05).

**Conclusion:** High glucose induced endothelial dysfunction and sulfasalazine reduced damage in high glucose vessels by activating eNOS, antioxidant effect through HO-1 enzymes and particularly inducing Nrf2 *via* the ERK and JNK pathways.

## Introduction

Diabetes mellitus is a disease that leads to various pathologies in several organs. Hyperglycemia induces endothelial dysfunction and contractile-relaxation responses (Rodríguez-Mañas et al., 2003; Hemling et al., 2020), resulting from decreased nitric oxide synthesis and accumulation of endothelium-reactive oxygen radicals (Aiko et al., 1998; Reyes-Toso et al., 2002; Lespagnol et al., 2020). Furthermore, high glucose reduces the endothelium-mediated acetylcholine relaxation response (Akther et al., 2021). It has been found an increase in advanced glycation end-products (AGEs) under diabetic conditions associated with endothelial dysfunction and vascular inflammation (Stirban et al., 2006; Soro-Paavonen et al., 2010; Ren et al., 2017). Under hyperglycemic conditions AGEs were able to decrease NO production and eNOS expression (Soro-Paavonen et al., 2010). AGEs also increase ROS production by activating NADPH oxidase (Zhuang et al., 2012).

The production of AGEs activates a numerous signaling pathways including mitogen-activating protein kinase (MAPK), extracellular signal-regulated kinase (ERK) Janus kinase family (JNK) and Nuclear Factor kappa B (NF-κB) leading to enhanced oxidative stress by producing pro-inflammatory factors including tumor necrosis factor (TNF) α, interleukin (IL)-1β, IL-8, and IL-6 (Liang et al., 2018; Khalid et al., 2022). Nuclear factor erythroid-2-related factor 2 (Nrf2) is a transcription factor crucial in maintaining the intracellular redox status by regulating the expression of antioxidant enzymes such as heme oxygenase—1 (HO-1) and many essential genes (Nguyen et al., 2000; Zhu and Fahl, 2001; Huang et al., 2015; Loboda et al., 2016; Seminotti et al., 2021; Zhang et al., 2021).

Various studies have addressed therapies targeting the (Nrf2)/Kelch-like ECH-related protein 1 (Keap1)/Antioxidant Response (ARE) pathway is the principal inducible defense against oxidative stresses in various disease including diabetes mellitus (Baird and Yamamoto, 2020). It has been found that the overexpression of KEAP1 was shown to suppress the nuclear accumulation and transcriptional activity of NRF2 (Baird and Yamamoto, 2020). Nrf2 deficiency exacerbates hyperglycemia and cellular redox state in types I and type II diabetes (Aleksunes et al., 2010; Bitar and Al-Mulla, 2011; Korac et al., 2021). Similarly a recent study showed that high glucose concentrations lead to alteration of the redox regulatory capacity of Nrf2 mediated by NF-kB regulation (Albert-Garay et al., 2022). These oxygen radicals accumulate in the endothelium, causing damage to materials such as cell membrane, DNA, enzymes, and decreased nitric oxide synthesis (De Vriese et al., 2000; Devasagayam et al., 2004). Nrf2 seems to be the dominant mechanism to clear reactive oxygen radicals formed by hyperglycemia. In addition, the Nrf2/Keap1/ARE pathway has long-term antioxidant properties more effective than other defensive mechanisms (Dinkova-Kostova and Talalay, 2008). Under stress conditions, Keap1 represses the enzymatic activity (Tebay et al., 2015) of Nrf2. Thus, free Nrf2 moves to the nucleus and binds to ARE, which encodes cytoprotective, antioxidant, and phase II detoxifying enzymes, for example, NAD(P)H: quinone reductase-1 (NQO1) and heme oxygenase-1 (HO-1) (Tebay et al., 2015). Furthermore, another study depicted the antioxidant effects by activating the Nrf2 signaling pathway and inducing the ARE-regulated expression of its downstream target genes, such as HO-1 (Serafini et al., 2019).

Sulfasalazine is an old drug used to treat chronic inflammatory diseases such as ulcerative colitis and rheumatoid arthritis. Mechanisms of its anti-inflammatory and immunomodulatory effects are only partially understood (Rains et al., 1995; Gan et al., 2005; Choi and Fenando, 2022). However, a study on diabetes mellitus, demonstrated 5-aminosalicylic acid can induce Nrf2 activation by separating Keep one from Nrf2 (Kang et al., 2017). The sulfasalazine induces HO-1 *via* reactive oxygen species (ROS)-dependent Nrf2 signaling, thereby reducing reactive oxygen radicals and oxidative stress in vessel walls (Kim et al., 2009; Robertson et al., 2020).

This study was carried out to investigate the effects of sulfasalazine on endothelial dysfunction evoked by high glucose. In this study we used isolated organ bath experiments and molecular analysis to explain the mechanism of the glucose-induced endothelial dysfunction and the therapeutic potential of sulfasalazine.

## Materials and methods

### Animals and experimental groups

In this study, we used 80 male adult Wistar Albino rats, weighed 250–300 g. The rats were obtained from the Aziz Sancar Research Institute of Experimental Medicine, Istanbul University (DETAE) and randomly divided into control, mannitol control, high glucose, sulfasalazine, and ERK, and JNK inhibitor groups. Local ethical committee approval was also obtained from the Istanbul University, Aziz Sancar Local Experimental Animal Ethics Committee (Approval No.35980450-050.01.04).

### Experimental protocol

Rats were given 0.4 ml of ketamine (500 mg/10 ml commercial provider) intraperitoneally and became unconscious within 3–5 min. The rat was fixed and abdomen was opened to get the thoracic aorta. The thoracic aorta was taken to the petri dish containing the oxygenated Krebs solution. Krebs solution was injected into the removed aorta, and the blood in the lumen was evacuated to prevent clot formation. Approximately 3 mm sections were taken from the aorta, which were cleaned from fat and other tissues and were kept in the beaker containing 11.1 mM glucose for the control and 44 mM glucose high glucose groups for 2 h.

Rings were prepared from thoracic aorta and they were mounted between two stainless steel hooks. Next, they were suspended in a 10 ml organ bath containing Krebs’ solution, pH 7.4, containing 118 mM NaCl, 4.7 mM KCl, 2.5 mM CaCl_2_, 1.2 mM MgSO_4_7H_2_O, 25 mM NaHCO_3_, 11.1 mM Glucose and gassed with 95%O_2_/5%CO_2_. An initial load of 2.0 g was applied. The tension of the aortic ring was monitored by a force transducer and recorded in a data acquisition system for 45 min’ equilibration (changing physiological fluids every 15 min). For the high glucose and mannitol Krebs-Henseleit solution, we used an extra 44 mM glucose and 44 mM mannitol. Noradrenaline (stock solution 1 mM), acetylcholine HCl (stock solution 1 mM), sulfasalazine (stock solutions 300 and 600 mM), U0126 (stock solution 1 mM), SP600125 (stock solution 1 mM) and other biochemical component were obtained from Sigma Aldrich, United States (Zhu et al., 2020; Zhou et al., 2021; Awad et al., 2022).

The organ baths were filled with high-glucose Krebs solution for high-glucose groups, while other baths were filled with normal glucose. Concentration-response curves were measured by cumulatively increasing noradrenaline or acetylcholine in glucose-free Krebs solution.

### Organ bath

Aortic rings let get stabilized at 2 g of rest tension for 30 min before the study began. The vessels were mounted on isolated organ bath (4,050; Ugo-basile, Gemonio, Italy) with 25 ml KHS, ventilated at 37°C (95% molecular oxygen and 5% carbon dioxide). A force-displacement transducer (MP36; Biopack Systems Inc., Goleta, CA, United States ) and integrated Tissue Bath Sysytem (ITBS08, Commat Ltd., Ankara, Turkey) were used to analyze the isolated aortic rings isometrically (Cantürk et al., 2010).

To determine contraction responses, and endothelium-derived nitric oxide-mediated vasorelaxation, aortic rings were exposed to acetylcholine and noradrenaline.

Noradrenaline (NA) was applied cumulatively in half-log steps beginning with 1 nM up to 30 µM. After applying NA, the organ baths were washed at intervals of 15 min and filled with new Krebs solution to allow the strip to recover (Rodríguez-Mañas et al., 2003; Vogel, 2008; Mathiew et al., 2020).

After application of 3 nM sub-therapeutic dose of NA, cumulatively in half-log steps beginning with 1 nM up to 30 µM Acetylcholine. At the end of the experiment, the vessels in the organ bath were taken, and the wet weights were recorded for further calculations (Rodríguez-Mañas et al., 2003; Mathiew et al., 2020).

### Incubation of sulfasalazine and inhibitors (janus kinase family and extracellular signal-regulated kinase)

In this study we used two different concentration of sulfasalazine 300 and 600 mM. Aortic rings were pre-treated with high glucose (44 mM) or with 300 and 600 mM sulfasalazine for 2 h and 30 min. The vascular response of both concentration of sulfasalazine were same therefore we used the lowest concentration i.e., 300 mM for the combination treatment. Each inhibitor (JNK inhibitor 10^−5^ M; SP600125 and ERK inhibitor 10^−6^ M; U0126) was added to the organ bath 30 min before the addition of sulfasalazine in high glucose (44 mM) groups only. Inhibitor concentrations (JNK and ERK) were selected on the previously used concentration in similar studies (Ok et al., 2014; Yu et al., 2015; Kim et al., 2020).

After assessing vascular responses, sections of aortic rings were harvested, homogenized, and prepared for biochemical measurements.

### Homogenization

A Bead homogenizer was used for the tissue homogenization. Tissues were first cut into smaller sections and placed in Eppendorf tubes with beads and homogenized in 5% extraction buffer using a glass homogenizer. The homogenates were transferred to 1.5 ml Eppendorf tubes, centrifuged at 12,000 × g for 10 min at 4°C, and the supernatant was stored at −80°C until analyzed for ELISA measurements.

### ELISA

According to the manufacturer’s specifications, the sandwich—ELISA method was used for biochemical measurements (Elabscience Biotechnology, Houston, Texas, United States ). Standards and samples were added to the specified wells and incubated conjugated with specific antibodies. Washing was done to separate free components, and the substrates were added to the wells. The ratio between the sample and standard concentrations was taken into concentration. The color change was observed with a spectrometer at 450 nm wavelength.

### Biochemical parameters

Oxidative stress and antioxidant capacity were measured by total antioxidant capacity (TAS) and total oxidant capacity (TOS) in homogenates. Nrf2 activation was then assessed by measuring levels of heme oxygenase-1 (HO-1) enzymes. The nitric oxide synthase (eNOS) relaxation parameter was also measured.

### Statistical analysis

The contraction response was calculated as mg tension/mg vessel wet weight. Relaxation responses were calculated by taking the percentage of submaximal noradrenaline contraction. EC_50_ graphs were drawn from the contraction and relaxation dose-response curves to find pD_2_ and maximum contraction and relaxation response values. The data are expressed as the mean ± standard error and were analyzed by GraphPad Prism^®^ (Version 5.0; GraphPad Software, Inc., La Jolla, CA, United States ) software. Statistical comparisons were made using one-way, two-way ANOVA followed by Post-Hoc Tukey’s and Bonferroni’s multiple comparison test. At the same time, the Student t-test is used for inter-group comparison. *p* < 0.05 was considered to be statistically significant.

The “n” indicated in the experimental groups shows the number of vessel rings isolated from different animals. The contraction responses were normalized to tissue weight and given in “mg/mg.” Relaxation responses were measured in pre-contracted arteries and given as a percent of maximum relaxation responses with sodium nitroprusside. The maximal contraction or relaxation responses (E_max_) were taken from curve fit; pD_2_ referred to the concentration giving 50% of the maximum response (contraction or relaxation), i.e., the negative (−) logarithm of the EC50.

## Results

### Effects of high glucose in rat aorta

**FIGURE 1:**
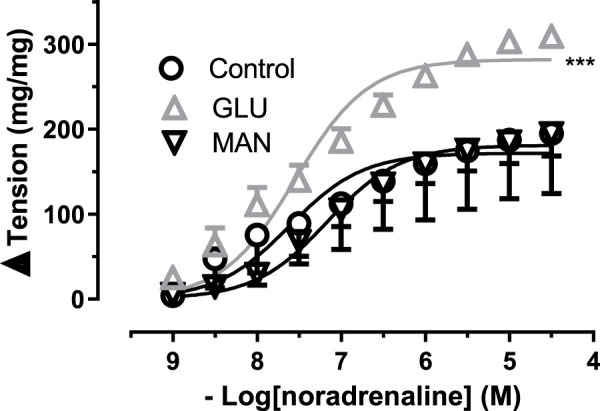
Effect of high glucose concentration on noradrenaline-induced tension in rat aortic rings. Concentration-response curve for cumulatively increasing concentrations of noradrenaline in the presence of physiological glucose concentration (11.1 mM, Control) and in the presence of 44 mM glucose (GLU) or 44 mM mannitol (MAN). Symbols indicate mean values ±SEM, from 8, 7 and 8 aortic rings (Control, GLU and MAN). ***p < 0.0001 for larger E_max_ in GLU vs. Control (F-test).

The contractile responses of the aortic rings to NA obtained in the presence of 44 mM mannitol (MAN) did not differ from the curve measured in physiological glucose concentration (11.1 mM, control). However, the 44 mM glucose (GLU) contraction curve enlarged significantly in Figure 1. Similarly, the E_max_ value was significantly larger in GLU than in the control and MAN groups, while pD_2_ values did not differ (Supplementary Table S1).

**FIGURE 2:**
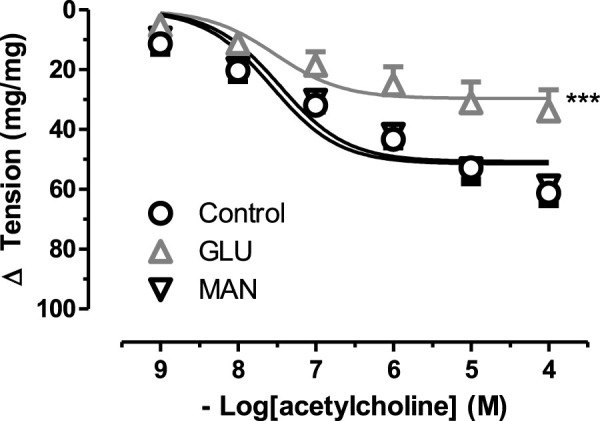
Effect of high glucose concentration on acetylcholine induce relaxation tension in rat aortic rings. Concentration-response curve for cumulatively increaseing concentrations of acetylcholine induced relatation in rat aortic rings, express as delta tension the presence of physiological glucose concentration (11.1 mM, Control), and in the presence of 44 mM glucose (GLU) or 44 mM mannitol (MAN). Symbols indicate mean values ±SEM, from 9, 12 and 8 aortic rings (Control, MAN and GLU). ***p < 0.0001 for larger E_max_ in GLU vs. Control (F-test).

To further test the effect of high glucose, we measured the relaxation tension in response to ACh. There was no significant difference in the 44 mM Mannitol (MAN) and control group (Figure 2), neither in E_max_ nor in pD_2_. However, in the GLU group, high glucose inhibited vascular relaxation in response to ACh compared to the control. Similarly, the E_max_ value was significantly smaller in the GLU group than in the control group and the respective pD_2_ values were significantly smaller. Furthermore, there was no significant difference according to the pD_2_ relaxation values of control groups (MAN, control) were compared (Supplementary Table S2).

**FIGURE 3:**
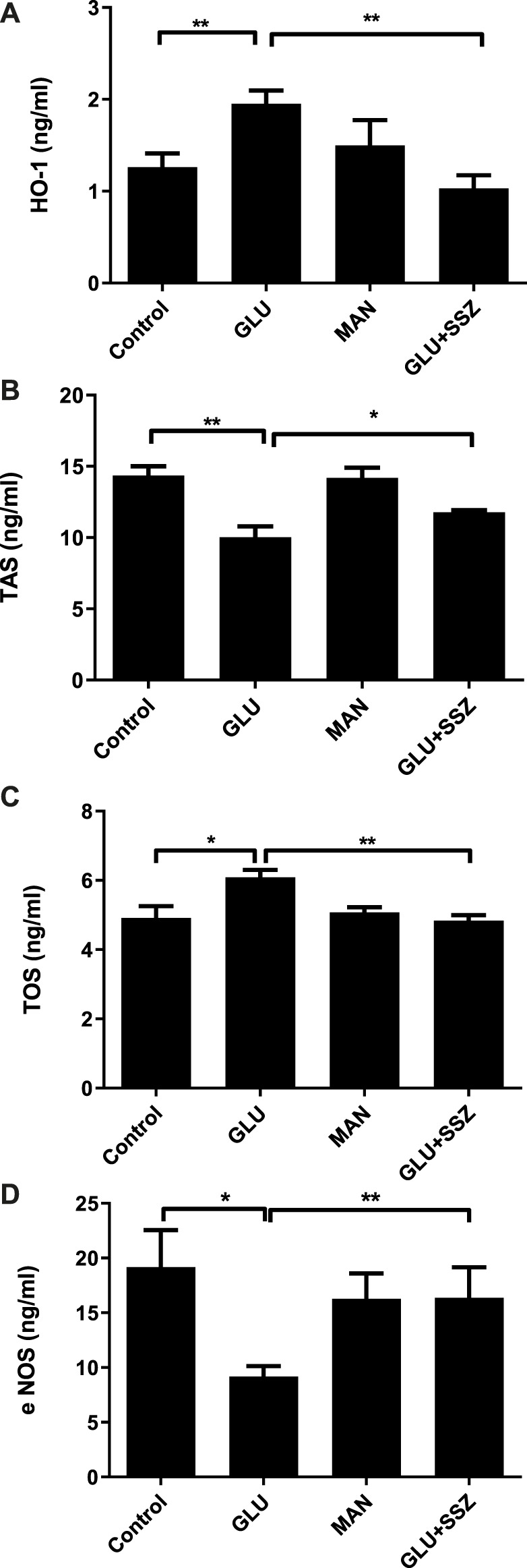
**(A)** Effect of sulfasalazine on the heme oxhgenase-1. The heme oxhgenase-1(HO-1) concentrations (ng/ml) in rat aortic rings, express the presence physiological glucose concentration (11.1 mM, Control), 44 mM glucose (GLU), 44 mM mannitol (MAN), 44 mM glucose and 300 mM sulfasalazine (GLU + SSZ). Bars indicate mean values ±SEM, from 9, 9, 6 and 5 aortic rings (Control, GLU, MAN, GLU + SSZ). **p < 0.001 for larger concentration HO-1 in GLU vs. Control and **p < 0.001 for smaller concentration GLU + SSZ vs. GLU (Unpaired *t* test). **(B)** Effect of sulfasalazine on the total antioxidant capacity. The total antioxidant capacity (TAS) concentrations (ng/ml) in rat aortic rings, express the presence physiological glucose concentration (11.1 mM, Control), 44 mM glucose (GLU), 44 mM mannitol (MAN), 44 mM glucose and 300 mM sulfasalazine (GLU + SSZ) Bars indicate mean values ±SEM, from 12, 4, 6 and 5 aortic rings (Control, GLU, MAN, GLU + SSZ). **p < 0.01 for smaller concentration TAS in GLU vs. Control and *p < 0.05 for larger concentration GLU + SSZ vs. GLU (Unpaired *t* test). **(C)** Effect of sulfasalazine on the total oxidant capacity. The total oxidant capacity (T0S) concentrations (ng/ml) in rat aortic rings, express the presence physiological glucose concentration (11.1 mM, Control), 44 mM glucose (GLU), 44 mM mannitol (MAN), 44 mM glucose and 300 mM sulfasalazine (GLU + SSZ) Bars indicate mean values ±SEM, from 5, 5, 5 and 5 aortic rings (Control, GLU, MAN, GLU + SSZ). *p < 0.05 for larger concentration TOS in GLU vs. Control and **p < 0.01 for smaller concentration GLU + SSZ vs. GLU (Unpaired *t* test). **(D)** Effect of sulfasalazine on the endothelial nitric oxide synthase. The endothelial nitric oxide synthase (eNOS) concentrations (ng/ml) in rat aortic rings, express the presence physiological glucose concentration (11.1 mM, Control), 44 mM glucose (GLU), 44 mM mannitol (MAN), 44 mM glucose and 300 mM sulfasalazine (GLU + SSZ) Bars indicate mean values ±SEM, from 12, 11, 6 and 6 aortic rings (Control, GLU, MAN, GLU + SSZ). *p < 0.05 for smaller concentration eNOS in GLU vs. Control and **p < 0.001 for larger concentration GLU + SSZ vs. GLU (Unpaired *t* test).

To extend our findings, we measured the HO-1 levels, the end product of the Nrf2 pathway, and we saw that its values in the high glucose group were significantly increased compared to the control group (Figure 3A, 1.95 ± 0.41 ng/ml *n* = 9 vs. 1.26 ± 0.42 ng/ml *n* = 9). Subsequently, the TAS values of the GLU group were significantly decreased compared to the control group (Figure 3B, 10.01 ± 1.32 ng/ml *n* = 4 vs.14.35 ± 2.19 ng/ml *n* = 12). As shown in Figure 3C, the TOS values of the high glucose group were significantly increased compared to the control group (Figure 3C, 6.08 ± 0.43 ng/ml *n* = 5 vs. 4.91 ± 0.67 ng/ml *n* = 5).

To explore the MAP kinase activation, we measured endothelial Nitric Oxide Synthase (eNOS) levels. The eNOS values of the GLU group were significantly smaller compared to the control group (Figure 3D, 9.17 ± 3.07 ng/ml *n* = 11 vs. 19.16 ± 11.625 ng/ml *n* = 12).

### Sulfasalazine reversed the high glucose-induced endothelial dysfunction

**FIGURE 4:**
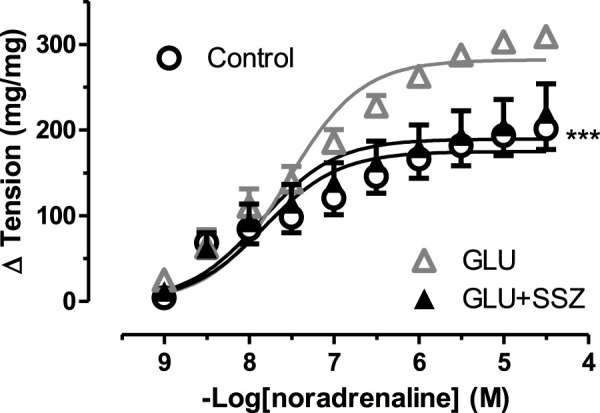
Effect of sulfasalazine on noradrenaline induce tension in rat aortic rings. Concentration-response curve for cumulatively increasing concentrations of noradrenaline in the presence of physiological glucose concentration (11.1 mM, Control), 44 mM glucose (GLU), 44 mM glucose and 300 mM sulfasalazine (GLU + SSZ). Symbols indicate mean values ±SEM, from 8, 7 and 8 aortic rings (Control, GLU and GLU + SSZ). ***p < 0.0001 for smaller E_max_ in GLU + SSZ vs. GLU (F-test).

To check the functional effect of sulfasalazine, the concentration-response curve for noradrenaline of 44 mM glucose and 300 mM sulfasalazine group (GLU + SSZ) compared to each other, and the sulfasalazine group showed a significant smaller again. The sulfasalazine group was no significant difference compared to the control group (Figure 4). Similarly, the E_max_ value of the sulfasalazine group was significantly smaller than the GLU group. The pD_2_ values of the sulfasalazine and the GLU groups were no significant difference (Supplementary Table S3).

**FIGURE 5:**
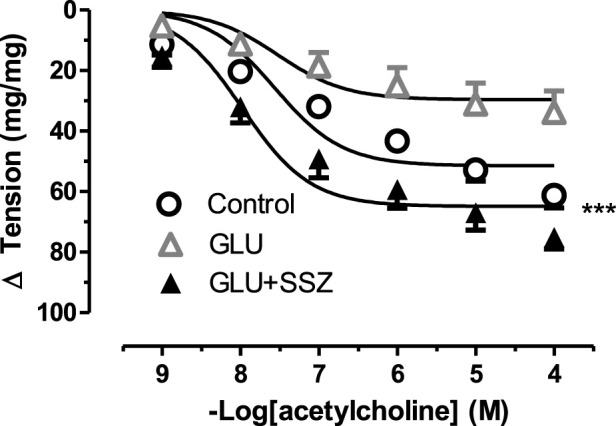
Effect of sulfasalazine on acetylcholine induce tension in rat aortic rings. Concentration-response curve for cumulatively increaseing concentrations of acetylcholine induced relatation in rat aortic rings, express as delta tension the presence of physiological glucose concentration (11.1 mM, Control), 44 mM glucose (GLU) and in the presence of 44 mM glucose and 300 mM sulfasalazine (GLU + SSZ). Symbols indicate mean values ±SEM, from 9, 12 and 11 aortic rings (Control, GLU and GLU + SSZ). ***p < 0.0001 for smaller E_max_ GLU + SSZ vs. Control (F-test).

To demonstrate relaxation response, the acetylcholine curve of the GLU and sulfasalazine groups was compared (Figure 5). The relaxation tension in the sulfasalazine group enlarged according to the GLU group. As shown in Figure 5, there was no significant difference comparing the sulfasalazine group with the control. Similarly, the E_max_ relaxation value of the sulfasalazine group was larger than the GLU group (Supplementary Table S4).

On the other hand, the HO—1 and TOS levels significantly decreased in the sulfasalazine group ompared to GLU (Figure 3A, 1.03 ± 0.28 ng/ml *n* = 5 vs. 1.95 ± 0.41 ng/ml *n* = 9; Figure 3C, 4.83 ± 0.31 ng/ml *n* = 5 vs. 6.08 ± 0.43 ng/ml *n* = 5). However, in the sulfasalazine group, TAS levels increased significantly compared to GLU (Figure 3B, 11.76 ± 0.29 ng/ml *n* = 5 vs. 10.01 ± 1.32 ng/ml *n* = 4). Similarly, eNOS levels increased significantly in the sulfasalazine group compared to GLU (Figure 3D, 16.37 ± 6.21 ng/ml *n* = 6 vs. 9.17 ± 3.07 ng/ml *n* = 11).

### Sulfasalazine activated the nuclear factor erythroid-2-related factor 2 in high glucose-induced vessels

**FIGURE 6:**
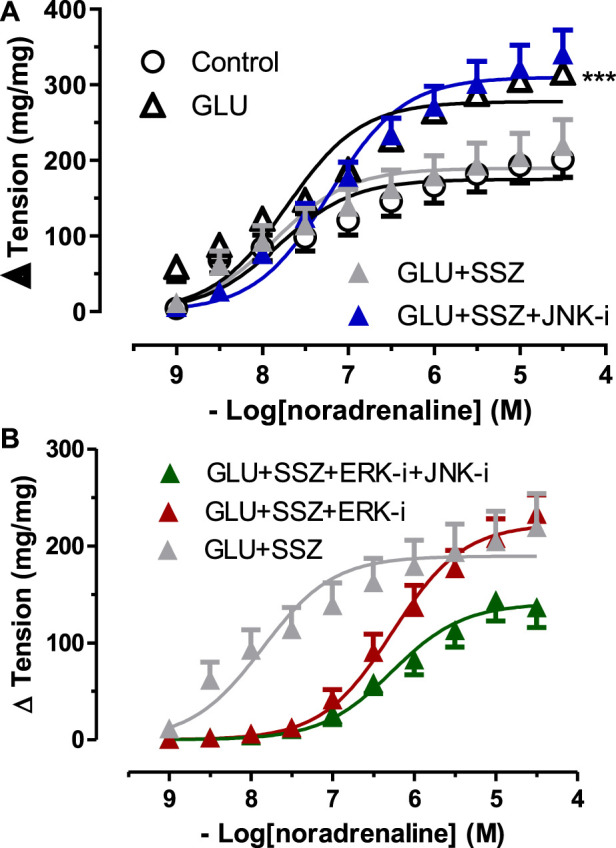
**(A)** Effect of JNK inhibitor on noradrenaline induce tension in rat aortic rings. Concentration-response curve for cumulatively increaseing concentrations of noradrenaline in the presence physiological glucose concentration (11.1 mM, Control), 44 mM glucose (GLU), 44 mM glucose and 300 mM sulfasalazine (GLU + SSZ), 44 mM glucose, 300 mM sulfasalazine and 10 µM JNK inhibitor SP600125 (GLU + SSZ + JNK-i). Symbols indicate mean values ±SEM, from 8, 7, 8 and 12 aortic rings (Control, GLU, GLU + SSZ, GLU + SSZ + JNK-i). ***p < 0.0001 for larger E_max_ in GLU + SSZ + JNK-i vs. GLU + SSZ (F-test). **(B)** Effect of JNK and ERK inhibitors on noradrenaline induce tension in rat aortic rings. Concentration-response curve for cumulatively increaseing concentrations of noradrenaline in the presence physiological glucose concentration (11.1 mM, control), 44 mM glucose (GLU), 44 mM glucose and 300 mM sulfasalazine (GLU + SSZ), 44 mM glucose, 300 mM sulfasalazine and 10 µM ERK inhibitor U0126 (GLU + SSZ + ERK-i), 44 mM glucose, 300 mM sulfasalazine, 10 µM ERK inhibitor U0126 and 10 µM JNK inhibitor SP600125 (GLU + SSZ + ERK-i + JNK-i).

To elucidate the effect of the ERK—JNK pathway on Nrf2, the contractile responses to NA was compared between the groups of sulfasalazine and inhibitor groups (10 µM JNK inhibitor SP600125, 10 µM ERK inhibitor U0126) (Figure 6A). In the JNK inhibitor group (GLU + SSZ + JNK-i), the contraction curve enhanced significantly compared to the sulfasalazine group (Figure 6B). The E_max_ value of the JNK group was larger than the sulfasalazine group. However, the E_max_ values of JNK and ERK inhibitors (GLU + SSZ + JNK-i + ERK-i) showed significantly smaller than the sulfasalazine group. (Supplementary Table S5).

**FIGURE 7:**
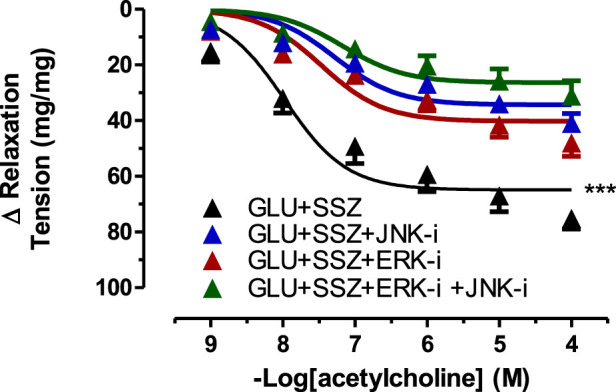
Effect of JNK and ERK inhibitors on acetylcholine induce tension in rat aortic rings. Concentration-response curve for cumulatively increaseing concentrations of acetylcholine induced relatation in rat aortic rings, express as delta tension the presence physiological glucose concentration (11.1 mM, Control), 44 mM glucose (GLU), 44 mM glucose and 300 mM sulfasalazine (GLU + SSZ), 44 mM glucose, 300 mM sulfasalazine and 10 µM JNK inhibitor SP600125 (GLU + SSZ + JNK-i), 44 mM glucose, 300 mM sulfasalazine and 10 mM ERK inhibitor U0126 (GLU + SSZ + ERK-i), 44 mM glucose, 300 mM sulfasalazine, 10 µM ERK inhibitor U0126 and 10 µM JNK inhibitor SP600125 (GLU + SSZ + ERK-i + JNK-i). Symbols indicate mean values ±SEM, from 9, 12, 11, 12, 11 and 10 aortic rings (Control, GLU, GLU + SSZ, GLU + SSZ + JNK-i, GLU + SSZ + ERK-i, GLU + SSZ + ERK-i + JNK i).***p < 0.0001 for smaller E_max_ in GLU + SSZ vs. GLU + SSZ + JNK-i, GLU + SSZ + ERK-i, GLU + SSZ + ERK-i + JNK-i (F-test).

Subsequently, it was shown that the inhibitor groups (JNK, ERK, and JNK + ERK) significantly inhibited vascular relaxation responses compared to the sulfasalazine group (Figure 7). The E_max_ values of inhibitor groups showed significantly smaller than the sulfasalazine group. There were no significant differences in the pD_2_ values of the inhibitors groups and the sulfasalazine group. (Supplementary Table S6).

**FIGURE 8:**
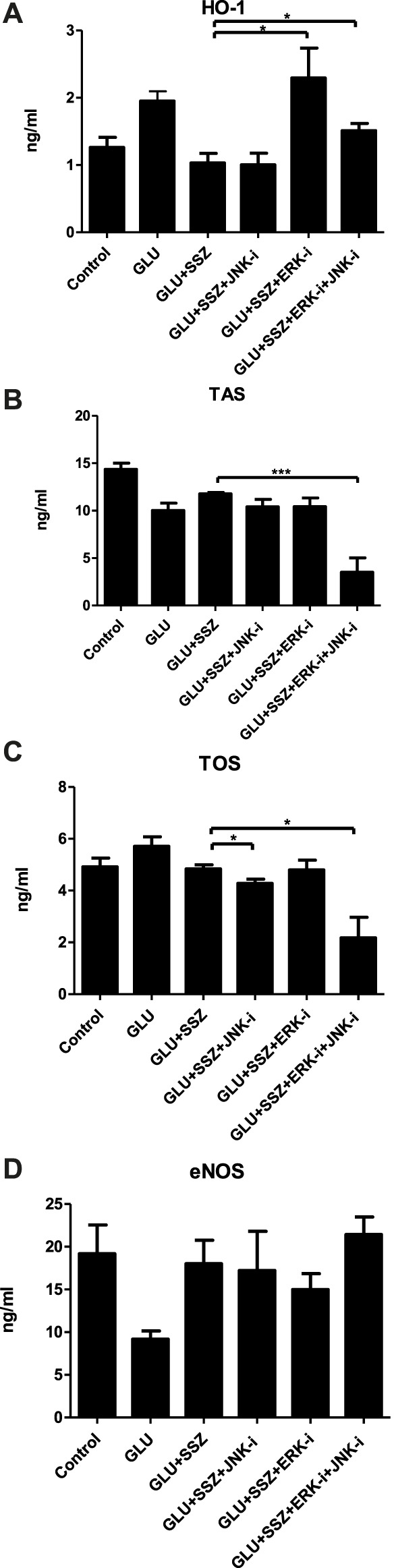
**(A)** Effect of ERK and JNK inhibitors on the heme oxhgenase-1. The heme oxhgenase-1(HO-1) concentrations (ng/ml) in rat aortic rings, express the presence physiological glucose concentration (11.1 mM, Control), 44 mM glucose (GLU), 44 mM glucose and 300 mM sulfasalazine (GLU + SSZ) 44 mM glucose, 300 mM sulfasalazine and 10 µM JNK inhibitor SP600125 GLU + SSZ + JNK-i), 44 mM glucose, 300 mM sulfasalazine, 10 µM ERK inhibitor U0126 (GLU + SSZ + ERK-i*), 44 mM glucose, 300 mM sulfasalazine, 10 µM ERK inhibitor U0126 and 10 µM JNK inhibitor SP600125 (GLU + SSZ + ERK-i + JNK-i). Bars indicate mean values ±SEM, from 9, 9, 5, 5, 5 and 5 aortic rings (Control, GLU, GLU + SSZ, GLU + SSZ + JNK-i. GLU + SSZ + ERK-i, GLU + SSZ + ERK-i + JNK-i). *p < 0.05 for larger concentration HO-1 in GLU + SSZ + ERK-i vs. GLU + SSZ and *p < 0.05 for smaller concentration GLU + SSZ + ERK-i + JNK-i vs. GLU + SSZ (Unpaired *t* test). **(B)** Effect of ERK and JNK inhibitors on total antioxidant capacity. The total antioxidant capacity (TAS) concentrations (ng/ml) in rat aortic rings, express the presence physiological glucose concentration (11.1 mM, Control), 44 mM glucose (GLU), 44 mM glucose and 300 mM sulfasalazine (GLU + SSZ) 44 mM glucose, 300 mM sulfasalazine and 10 µM JNK inhibitor SP600125 GLU + SSZ + JNK-i), 44 mM glucose, 300 mM sulfasalazine, 10 µM ERK inhibitor U0126 (GLU + SSZ + ERK-i*), 44 mM glucose, 300 mM sulfasalazine, 10 µM ERK inhibitor U0126 and 10 µM JNK inhibitor SP600125 (GLU + SSZ + ERK-i + JNK-i). Bars indicate mean values ±SEM, from 12, 4, 5, 4, 5 and 6 aortic rings (Control, GLU, GLU + SSZ, GLU + SSZ + JNK-i. GLU + SSZ + ERK-i, GLU + SSZ + ERK-i + JNK-i). ***p < 0.001 for smaller concentration TAS in GLU + SSZ + ERK-i + JNK-i vs. GLU + SSZ (Unpaired *t* test). **(C)** Effect of ERK and JNK inhibitors on total oxidant capacity. The total antioxidant capacity (TOS) concentrations (ng/ml) in rat aortic rings, express the presence physiological glucose concentration (11.1 mM, Control), 44 mM glucose (GLU), 44 mM glucose and 300 mM sulfasalazine (GLU + SSZ) 44 mM glucose, 300 mM sulfasalazine and 10 µM JNK inhibitor SP600125 GLU + SSZ + JNK-i), 44 mM glucose, 300 mM sulfasalazine, 10 µM ERK inhibitor U0126 (GLU + SSZ + ERK-i*), 44 mM glucose, 300 mM sulfasalazine, 10 µM ERK inhibitor U0126 and 10 µM JNK inhibitor SP600125 (GLU + SSZ + ERK-i + JNK-i). Bars indicate mean values ±SEM, from 12, 4, 5, 4, 5 and 6 aortic rings (Control, GLU, GLU + SSZ, GLU + SSZ + JNK-i. GLU + SSZ + ERK-i, GLU + SSZ + ERK-i + JNK-i). *p < 0.05 for smaller concentration TOS in GLU + SSZ + ERK-i + JNK-i vs. GLU + SSZ *p < 0.05 for smaller concentration GLU + SSZ + ERK-i + JNK-i vs. GLU + SSZ (Unpaired *t* test). **(D)** Effect of ERK and JNK inhibitors on endothelial nitric oxide synthase. The endothelial nitric oxide synthase (eNOS) concentrations (ng/ml) in rat aortic rings, express the presence physiological glucose concentration (11.1 mM, Control), 44 mM glucose (GLU), 44 mM glucose and 300 mM sulfasalazine (GLU + SSZ) 44 mM glucose, 300 mM sulfasalazine and 10 µM JNK inhibitor SP600125 GLU + SSZ + JNK-i), 44 mM glucose, 300 mM sulfasalazine, 10 µM ERK inhibitor U0126 (GLU + SSZ + ERK-i*), 44 mM glucose, 300 mM sulfasalazine, 10 µM ERK inhibitor U0126 and 10 µM JNK inhibitor SP600125 (GLU + SSZ + ERK-i + JNK-i). Bars indicate mean values ±SEM, from 12, 11, 5, 5, 5 and 5 aortic rings (Control, GLU, GLU + SSZ, GLU + SSZ + JNK-i. GLU + SSZ + ERK-i, GLU + SSZ + ERK-i + JNK-i).

Furthermore, HO-1 levels increased again in the ERK and ERK + JNK inhibitors groups compared to the sulfasalazine group (Figure 8A, 2.29 ± 0.88 ng/ml *n* = 5, 1.51 ± 0.21 ng/ml *n* = 5 vs. 1.03 ± 0.28 ng/ml *n* = 5). There was no significant difference comparing the JNK inhibitor group with the sulfasalazine group (Figure 8A). Similarly, the TAS and TOS levels were no significant changes in ERK and JNK inhibitor groups compared to the sulfasalazine group (Figures 8B,C). However, in JNK and ERK + JNK inhibitor groups, TOS levels significantly decreased compared to the sulfasalazine group (Figure 8C, 4.27 ± 0.33 ng/ml *n* = 5, 2.17 ± 1.58 ng/ml *n* = 5 vs. 4.83 ± 0.31 ng/ml *n* = 5). The eNOS levels also were no significant changes in the measured values in the cases of inhibitor groups compared to the sulfasalazine group (Figure 8D).

## Discussion

In this study, the high glucose-induced vascular dysfunction was demonstrated through the contractile responses of the aortic rings to NA and relaxation tension in response to Ach. Subsequently, sulfasalazine reversed high glucose-induced vascular constriction, increased eNOS, and decreased TOS and HO-1. Finally, to investigate the mechanism of action sulfasalazine on the treatment of vascular dysfunction we used the JNK and ERK inhibitors and the data revealed that these inhibitors partially prevented the sulfasalazine effect in vessels.

High glucose induces oxidative stress on vessels can cause endothelial dysfunction (Tian et al., 2014; Goulopoulou et al., 2015; Kaur et al., 2022). In streptozotocin-induced-diabetic vessels of rats, the contractile response was greater as compared to control (Majithiya and Balaraman, 2005; Baylan et al., 2022). However, in another study with streptozotocin-induced -diabetic rats, the vessels contractions were smaller in comparison to control (Lingbo et al., 2005). We demonstrated that high glucose increased the contraction responses to NA compared to the control. In line with our study, the acute effect of exposure to high glucose showed an increased vascular contraction in mice aorta (Xu et al., 2021).

Conversely, we found significant decrease in relaxation response of vessels of high glucose group. Which is line with previous studies as many researchers showed that high glucose incubation reduced vessel relaxation (Pieper et al., 1997; Guo et al., 2000; Miike et al., 2008). The literature attributes these deteriorations in vascular responses to endothelial dysfunction resulting from the accumulation of oxygen radicals (De Vriese et al., 2000; Capellini et al., 2010).

In line with an earlier *in vitro* study (Steel et al., 2012), we found a significant increase in HO-1 concentration in high glucose. Similarly, HO-1 expression increased in diabetic rats and this increase could improve impaired vascular endothelium (Mingone et al., 2008). The vasculature has a abundance evidence of protective effect against oxidation and inflammation, most of them regulated by the Nrf2 pathway. HO-1 is a Nrf2-regulated gene that plays a major role in the inhibition of vascular inflammation ((Araujo et al., 2012). Therefore, high HO-1 levels could be an antioxidant defensive response resulting from the activation of Nrf2 by high glucose. Similarly, in a latest study conducted by Yao et al. (2020) kaempferol protects blood vessels from damage in association with Nrf2/HO-1 signaling pathway. This assumption is supported by the finding that Nrf2 deficiency might accelerate the progression of type I and type II diabetes (Aleksunes et al., 2010; Bitar and Al-Mulla, 2011; Gutiérrez-Cuevas et al., 2022). In previous studies, it was reported that TAS copes with oxidative stress (Devasagayam et al., 2004; Strycharz-Dudziak et al., 2019). However, in our study, TAS levels were significantly decreased in the high glucose group. According to this result, we suggested that TAS antioxidant mechanisms could not prevent oxidative stress in our high glucose group. In a study conducted by Tota et al. (2021) on 30 type 1 DM patients found that the TOS/TAS and nitrosative stress indicators were significantly higher in T1DM. TOS/TAS marker is measuring plasma antioxidant capacity, represent the redox state in the body and is more in a more operational than the evaluation of a single circulating antioxidant (Erel, 2005). In addition, high glucose causes damage to endothelial cells by increasing TOS concentrations and impairs vascular responses (Stocker and Keaney, 2004; Tota et al., 2021). This deterioration in vascular responses has also been attributed to NO-dependent mechanisms (Alp et al., 2003; Cai et al., 2005; Gaynullina et al., 2022). Therefore, we measured eNOS levels in the high glucose group and found a significantly decreased eNOS, parallel with other studies (Han et al., 2014; Kassan et al., 2014).

To show sulfasalazine antioxidant properties on the endothelium (Lodowska et al., 2015) and to explain nitric oxide synthesis in high glucose (Aiko et al., 1998), we measured HO-1 and eNOS levels. In our study, sulfasalazine increased again decreased HO-1 and eNOS levels, similar to the previous study (Aiko et al., 1998). Furthermore, we found impaired vessel responses with high glucose, and sulfasalazine improved contractile and relaxation responses. It suggests that sulfasalazine plays a role in ameliorating endothelial dysfunction through eNOS and HO-1.

We used ERK and JNK inhibitors in the presence of high glucose and sulfasalazine to investigate more in detail the mechanism of sulfasalazine action on Nrf2. In the presence of JNK inhibitor was found an enlarged again the contraction response to NA. Kim et al. (2009) showed that sulfasalazine activates Nrf2 *via* the JNK pathway in a ROS-dependent manner. Furthermore, sulfasalazine causes nuclear factor “kappa-light-chain-enhancer” of activated B-cells (Nf-kB) inhibition and indirectly provides JNK activation (Zhou et al., 2007). Our study suggests that sulfasalazine activates the JNK pathway. We also found that TOS decreased in the JNK inhibitor, which may also occur because of Nf-kB activation.

In the presence of an ERK inhibitor, E_max_ for ACh-induced relaxation is significantly decreased and HO-1 levels increased again. Since previous studies reported that sulfasalazine induces HO-1 induction due to oxidative stress (Kim et al., 2009) and activates the ERK pathway (Han et al., 2014). Therefore, we suspected that sulfasalazine might provide Nrf2 activation *via* the ERK pathway. To extend our findings, we repeated the experiments in the concomitant presence of inhibitors of ERK and JNK together and reported that HO-1 levels increased significantly. Our data are in line with a recent study where high glucose activated Nrf2 and activating ROS-dependent JNK and ERK pathways increased HO-1 expression (Yang et al., 2016).

**FIGURE 9:**
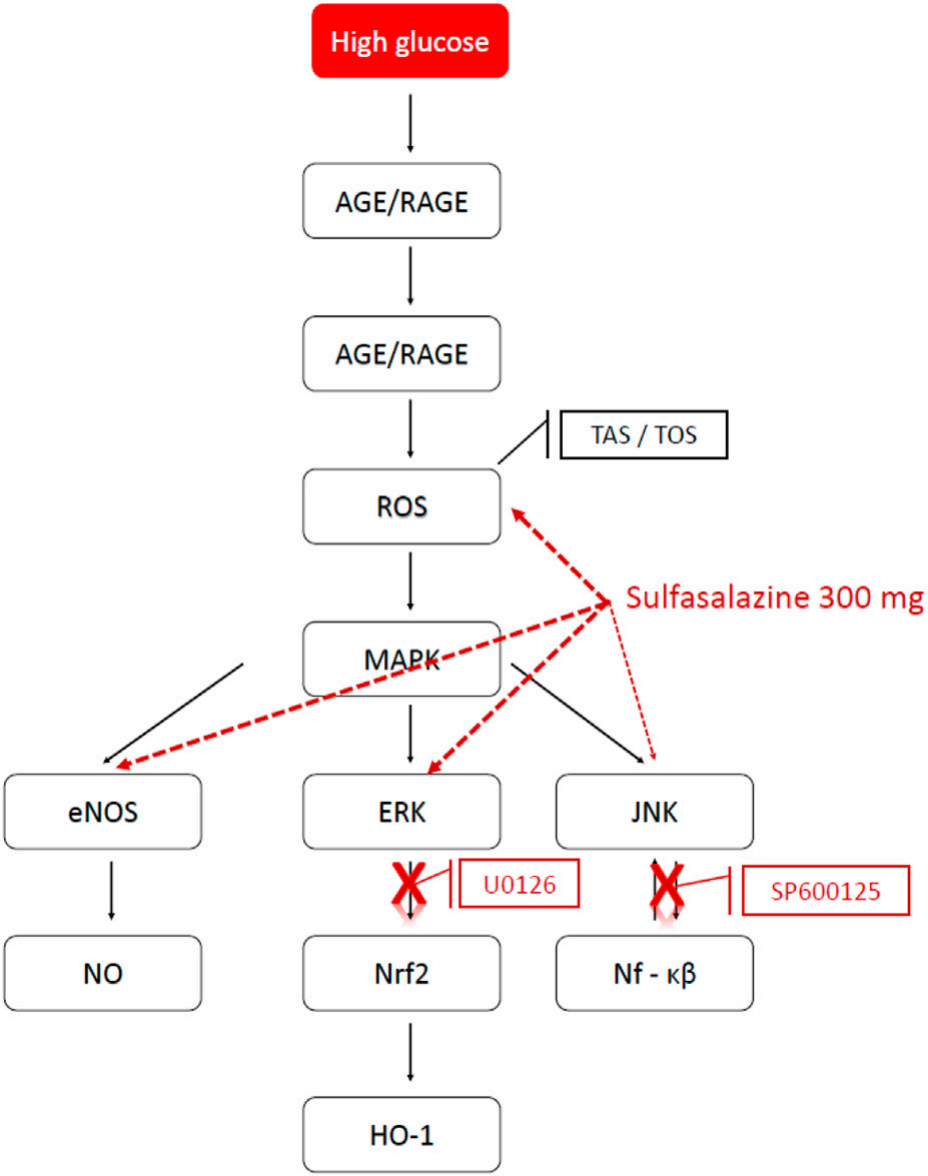
Possible pathways of action of sulfasalazine in high glucose. The possible pathways of action of sulfasalazine on endothelial dysfunction induced by high glucose. AGE, advanced glycation endproduct; RAGE, the receptor for advanced glycation endproduct; ROS, reactive oxygen speices; MAPK, the mitogen-activating protein kinase; eNOS, endothelial nitric oxide synthase; NO, nitric oxide; ERK, extracellular signal-regulated kinase; JNK, Janus kinase family; Nrf2, nuclear factor erythroid 2-related factor 2; Nf-kB, nuclear factor “kappa-light-chain-enhancer” of activated B-cells; HO-1, Heme oxygenase; U0126, ERK inhibitor; SP600125, JNK inhibitor (Prepared on 11.06.2018 using the website http://www.genome.jp/kegg/pathway.html).

In conclusion, we found that sulfasalazine activates on Nrf2 by ERK pathway, as shown through vascular responses and decreased HO-1 concentration. However, the JNK pathway was only activated in vascular response with no change in HO-1 concentration. Through activated eNOS, sulfasalazine could play a role in ameliorating endothelial dysfunction (Figure 9).

## Data Availability

The original contributions presented in the study are included in the article/Supplementary Materials, further inquiries can be directed to the corresponding author.

## Supplementary material

SUPPLEMENTARY TABLE S1 The pD_2_ and E_max_ values for contraction to noradrenaline of rat aorta.

SUPPLEMENTARY TABLE S2 The pD_2_ and E_max_ values for relaxation to acetylcholine of rat aorta.

SUPPLEMENTARY TABLE S3 The pD_2_ and E_max_ values for contraction to noradrenaline of rat aorta.

SUPPLEMENTARY TABLE S4 The pD_2_ and E_max_ values for relaxation to acetylcholine of rat aorta.

SUPPLEMENTARY TABLE S5 The pD_2_ and E_max_ values for contraction to noradrenaline of rat aorta.

SUPPLEMENTARY TABLE S6 The pD_2_ and E_max_ values for relaxation to acetylcholine of rat aorta.
