# Supplementary material for: Effect of sulfasalazine on endothelium-dependent vascular response by the activation of Nrf2 signalling pathway
Source: Front Pharmacol. 2022 Oct 24;13:979300. doi: 10.3389/fphar.2022.979300 (PMC9639785; doi:10.3389/fphar.2022.979300)
Supplement: Supplementary file 2 [file Table2.docx]

**Supplements**

| **Table 2 : The pD_2_ and E_max_ values** **for relaxation to acetylcholine of rat aorta** |
| --- |
| \|  \| **E_max_ (mg/mg)** \| **pD_2_** \| **n** \| \| --- \| --- \| --- \| --- \| \| **Control** \| 51,48± 2,48 \| 7,56± 0,15 \| 9 \| \| **GLU** \| 29,62± 3,02*** \| 7,52± 0,32 \| 12 \| \| **MAN** \| 50.71± 2,95 \| 7,46± 0,18 \| 8 \|   Maximum contractions (E_max_ ) (mg tension/mg aorta) and sensitivity (pD_2_ ) values to acethylcholine. Physiological glucose concentration (11.1 mM, Control) and in the presence of 44 mM glucose (GLU) or 44 mM mannitol (MAN). The “n” indicates the aortic rings (Control, GLU and MAN). *** p<0.0001 for larger E_max_ in GLU vs. Control (F-test). |
