# Supplementary material for: Effect of sulfasalazine on endothelium-dependent vascular response by the activation of Nrf2 signalling pathway
Source: Front Pharmacol. 2022 Oct 24;13:979300. doi: 10.3389/fphar.2022.979300 (PMC9639785; doi:10.3389/fphar.2022.979300)
Supplement: Supplementary file 3 [file Table5.docx]

**Supplements**

**Table 5 : The pD_2_ and E_max_ values** **for contraction to noradrenaline of rat aorta**

|  | **E_max_ (mg/mg)** | **pD_2_** | **n** |
| --- | --- | --- | --- |
| **GLU + SSZ** | 189,5± 10,85 | 7,85± 0,16 | 8 |
| **GLU+SSZ+JNK-i** | 310,1± 11,56 *** | 7,22± 0,09 | 12 |
| **GLU+SSZ+ERK-i** | 222,7± 10,14 | 6,26± 0,09 | 12 |
| **GLU+SSZ+JNK-i +ERK-i** | 140,7± 9,17 | 6,27± 0,13 | 11 |

Maximum contractions (E_max_ ) (mg tension/mg aorta) and sensitivity (pD_2_ ) values to noradrenaline. The 44 mM glucose and 300 mM sulfasalazine group (GLU+SSZ), the 44 mM glucose,300 mM sulfasalazine, and 10 µM JNK inhibitor SP600125 group (GLU+SSZ+JNK-i), the 44 mM glucose,300 mM sulfasalazine, and 10 µM ERK inhibitor U0126 group (GLU+SSZ+ERK-i), and the 44 mM glucose,300 mM sulfasalazine, 10 µM JNK inhibitor SP600125, and 10 µM ERK inhibitor U0126 group (GLU+SSZ+ERK-i) group (GLU+SSZ+JNK-i+ERK-i). The “n” indicates the aortic rings (GLU+SSZ and GLU + SSZ+JNK-i). *** p<0.0001 for larger E_max_ in GLU vs. Control (F-test).
