# Supplementary material for: Effect of sulfasalazine on endothelium-dependent vascular response by the activation of Nrf2 signalling pathway
Source: Front Pharmacol. 2022 Oct 24;13:979300. doi: 10.3389/fphar.2022.979300 (PMC9639785; doi:10.3389/fphar.2022.979300)
Supplement: Supplementary file 4 [file Table3.docx]

**Supplements**

**Table 3 : The pD_2_ and E_max_ values** **for contraction to noradrenaline of rat aorta**

|  | **E_max_ (mg/mg)** | **pD_2_** | **n** |
| --- | --- | --- | --- |
| **GLU** | 282,0 ± 7,8 | 7,54 ± 0,07 | 7 |
| **GLU+SSZ** | 189,5± 10,85** | 7,85± 0,16 | 8 |

Maximum contractions (E_max_ ) (mg tension/mg aorta) and sensitivity (pD_2_ ) values to noradrenaline. The presence of 44 mM glucose (GLU) and 300 mM sulfasalazine group (GLU+SSZ). The “n” indicates the aortic rings (GLU and GLU + SSZ). ** p<0.001 for larger E_max_ in GLU vs. GLU+SSZ (F-test).
