# Supplementary material for: Effect of sulfasalazine on endothelium-dependent vascular response by the activation of Nrf2 signalling pathway
Source: Front Pharmacol. 2022 Oct 24;13:979300. doi: 10.3389/fphar.2022.979300 (PMC9639785; doi:10.3389/fphar.2022.979300)
Supplement: Supplementary file 5 [file Table4.docx]

**Supplements**

**Table 4 : The pD_2_ and E_max_ values** **for relaxation to acetylcholine of rat aorta**

|  | **E_max_ (mg/mg)** | **pD_2_** | **n** |
| --- | --- | --- | --- |
| **GLU** | 29,62± 3,02 | 7,52± 0,32 | 12 |
| **GLU+SSZ** | 64,91± 3,04*** | 7,97± 0,14 | 11 |

Maximum contractions (E_max_ ) (mg tension/mg aorta) and sensitivity (pD_2_ ) values to acetylcholine. The presence of 44 mM glucose (GLU) and 300 mM sulfasalazine group (GLU+SSZ). The “n” indicates the aortic rings (GLU and GLU + SSZ). *** p<0.0001 for larger E_max_ in GLU vs. GLU+SSZ (F-test).
