# Supplementary material for: Effect of sulfasalazine on endothelium-dependent vascular response by the activation of Nrf2 signalling pathway
Source: Front Pharmacol. 2022 Oct 24;13:979300. doi: 10.3389/fphar.2022.979300 (PMC9639785; doi:10.3389/fphar.2022.979300)
Supplement: Supplementary file 6 [file Table6.docx]

**Supplements**

Table 6: The pD_2_ and E_max_ values for relaxation to acetylcholine of rat aorta

|  | **E_max_ (mg/mg)** | **pD_2_** | **n** |
| --- | --- | --- | --- |
| **GLU + SSZ** | 64,91± 3,04*** | 7,97± 0,14 | 11 |
| **GLU+SSZ+JNK-i** | 34,33 ± 1,63 | 7,28± 0,14 | 12 |
| **GLU+SSZ+ERK-i** | 40,240± 2,35 | 7,46± 0,18 | 11 |
| **GLU+SSZ+JNK-i +ERK-i** | 26,38± 0,23 | 7,17± 0,23 | 10 |

Maximum contractions (E_max_ ) (mg tension/mg aorta) and sensitivity (pD_2_ ) values to acetylcholine. The 44 mM glucose and 300 mM sulfasalazine group (GLU+SSZ), the 44 mM glucose,300 mM sulfasalazine, and 10 µM JNK inhibitor SP600125 group (GLU+SSZ+JNK-i), the 44 mM glucose,300 mM sulfasalazine, and 10 µM ERK inhibitor U0126 group (GLU+SSZ+ERK-i), and the 44 mM glucose,300 mM sulfasalazine, 10 µM JNK inhibitor SP600125, and 10 µM ERK inhibitor U0126 group (GLU+SSZ+ERK-i) group (GLU+SSZ+JNK-i+ERK-i). The “n” indicates the aortic rings (GLU+SSZ and GLU + SSZ+JNK-i). *** p<0.0001 for larger E_max_ in GLU vs. Control (F-test).
