# Supplementary material for: Effect of sulfasalazine on endothelium-dependent vascular response by the activation of Nrf2 signalling pathway
Source: Front Pharmacol. 2022 Oct 24;13:979300. doi: 10.3389/fphar.2022.979300 (PMC9639785; doi:10.3389/fphar.2022.979300)
Supplement: Supplementary file 7 [file DataSheet1.docx]

(Tebay et al., 2015)

Serafini, M.M., Catanzaro, M., Fagiani, F., Simoni, E., Caporaso, R., Dacrema, M., Romanoni, I., Govoni, S., Racchi, M., Daglia, M., Rosini, M., and Lanni, C. (2019). Modulation of Keap1/Nrf2/ARE Signaling Pathway by Curcuma- and Garlic-Derived Hybrids. *Front Pharmacol* 10**,** 1597.

Tebay, L.E., Robertson, H., Durant, S.T., Vitale, S.R., Penning, T.M., Dinkova-Kostova, A.T., and Hayes, J.D. (2015). Mechanisms of activation of the transcription factor Nrf2 by redox stressors, nutrient cues, and energy status and the pathways through which it attenuates degenerative disease. *Free Radical Biology and Medicine* 88**,** 108-146.

(Serafini et al., 2019)
